# Improving Well-being With a Mobile Artificial Intelligence–Powered Acceptance Commitment Therapy Tool: Pragmatic Retrospective Study

**DOI:** 10.2196/36018

**Published:** 2022-07-12

**Authors:** Navot Naor, Alex Frenkel, Mirène Winsberg

**Affiliations:** 1 Kai.ai Tel Aviv Israel; 2 Kai.ai San Francisco, CA United States

**Keywords:** Acceptance Commitment Therapy, well-being, WHO-5, World Health Organization-Five Well-Being Index, mHealth, mobile health, smartphone, health application, mental health, quality of life, artificial intelligence

## Abstract

**Background:**

Research and dissemination of smartphone apps to deliver coaching and psychological driven intervention had seen a great surge in recent years. Notably, Acceptance Commitment Therapy (ACT) protocols were shown to be uniquely effective in treating symptoms for both depression and anxiety when delivered through smartphone apps. The aim of this study is to expand on that work and test the suitability of artificial intelligence–driven interventions delivered directly through popular texting apps.

**Objective:**

This study evaluated our hypothesis that using Kai.ai will result in improved well-being.

**Methods:**

We performed a pragmatic retrospective analysis of 2909 users who used Kai.ai on one of the top messaging apps (iMessage, WhatsApp, Discord, Telegram, etc). Users’ well-being levels were tracked using the World Health Organization-Five Well-Being Index throughout the engagement with service. A 1-tailed paired samples *t* test was used to assess well-being levels before and after usage, and hierarchical linear modeling was used to examine the change in symptoms over time.

**Results:**

The median well-being score at the last measurement was higher (median 52) than that at the start of the intervention (median 40), indicating a significant improvement (W=2682927; *P*<.001). Furthermore, HLM results showed that the improvement in well-being was linearly related to the number of daily messages a user sent (β=.029; *t*_81.36_=4; *P*<.001), as well as the interaction between the number of messages and unique number of days (β=–.0003; *t*_81.36_=–2.2; *P*=.03).

**Conclusions:**

Mobile-based ACT interventions are effective means to improve individuals’ well-being. Our findings further demonstrate Kai.ai’s great promise in helping individuals improve and maintain high levels of well-being and thus improve their daily lives.

## Introduction

Psychological distress, which can be broadly understood as an emotional state of suffering from mood- and anxiety-related symptoms [1], is among the leading causes of disability in the world [2]. It has become highly prevalent; it was recently estimated that up to 50% of the world's population experiences symptoms of psychological distress [3]. Knowing how widespread mental health issues are, it is not surprising that they were found to be a central cause for the global burden of diseases worldwide, accounting for some 56.7% of disability-adjusted life years [2]. Furthermore, a recent report estimated that in 2010, poor mental health resulted in a US $2-$5 trillion annual loss worldwide owing to poor health and reduced productivity. This loss was only projected to increase, expected to approach US $6 trillion by 2030 [4].

While the COVID-19 pandemic imposed numerous hardships on the world, the increase in prevalence and severity of mental distress symptoms reached a level where worldwide organizations declared it a global crisis that needed immediate attention [4,5]. While the number of individuals with mental illnesses has been documented in the second decade of the 21st century, there was no comparable increase in the numbers of individuals receiving treatment for these symptoms over the same period. This highlights the unmet needs for more treatment possibilities [6]. Furthermore, it was recently claimed that despite the substantial investment in the development of new medications and therapeutic protocols, they are only expected to have a limited impact over the long term. In contrast, it is argued that greater attention should be given to the development of prevention strategies and tools [7].

Recently, mobile apps and computer-based applications have become increasingly useful tools for creating and disseminating mental health prevention and treatment programs [8-10]. Mobile interventions introduce a fresh variety of possibilities, including the provision for unlimited interactions and psychological interventions. This creates opportunities to reach patients anytime and anywhere. Furthermore, this kind of platform also has the possibility to enhance efficiency by increasing the treatment intensity and integrating therapeutic strategies into daily life [11].

The use of mobile-based innervations was found to be particularly effective when it was used to deliver Acceptance Commitment Therapy (ACT) protocols [12-15]. Since ACT protocols allow for a low-intensity or high-frequency approach [12], they are a highly effective method to facilitate changes in the flexibility of the thinking process, which is one of ACT’s central features [16]. ACT is a form of psychotherapy positioned within the third wave of behavioral therapies. It focuses on teaching patients how to deal with challenging experiences by reflecting on their values [16]. ACT works to develop psychological flexibility, which entails having the ability to stay in contact with the present regardless of any unpleasant thoughts, feelings, or bodily sensations. Recently, ACT was shown to be effective in treating symptoms of both depression [14] and anxiety [13].

In this pragmatic retrospective study, we report the test results of a mobile platform called Kai.ai. Kai.ai is a platform designed to provide ACT-based, artificial intelligence–driven conversational coaching that helps users build habits for healthy living and resilience. It integrates seamlessly with all the top messaging apps (iMessage, WhatsApp, Discord, Telegram, etc) allowing for ecological momentary assessment and ecological momentary interventions. The platform checks in with users throughout the day and leverages users’ responses and journaling exercises to deliver tailored ACT skilled coaching. In this study, the platform was evaluated using both a pre-post and longitudinal design to assess changes in measures of well-being among users. Our hypothesis being that using Kai.ai will result in improved well-being.

## Methods

### Study Design

A pragmatic retrospective design was used for this study, using data collected as part of users’ engagement with Kai.ai’s services. All participants were located in the United States and had free access to the service, which is seamlessly integrated with all the top messaging apps (iMessage, WhatsApp, Discord, Telegram, etc). This retrospective study included data from 2909 Kai.ai users, 85% of whom were recruited from major social networks such as TikTok, and they are presented with an advertisement inviting them to test how happy they are. Clicking the advertisement would direct users to a landing page (How happy are you? - Kai), where they can complete the World Health Organization-Five Well-Being Index (WHO-5). Upon completing the questionnaire, users were asked whether they would like to continue and engage with Kai.ai in the future.

During the first engagement, users were asked to complete an electronically secured version of the WHO-5. The survey was completely optional, and users could opt to not take part in the assessment phase. As users continued using Kai.ai, they were prompted once every 6 weeks to complete an additional WHO-5 assessment.

### Participants

Participants completed the WHO-5 at least twice, with the highest number of completed WHO-5 measurements being 9. All participants in this study were from the United States, but since the data set used in this study was completely anonymized before it was given to the authors, no gender- or age-related information was available.

### Ethical Considerations

This report is based on a postfactum analysis, which used anonymized data gathered during users’ engagement with Kai.ai’s services. As such, it was determined as not being a study involving human subjects by WCG IRB (1-1504102-1).

### WHO-5

The WHO-5 is a short self-report to measure well-being [17,18]. Items are rated on a 6-point Likert scale, where participants are asked to rate their experiences in the past 2 weeks from 5=all of the time to 0=at no time. Raw scores are then multiplied by 4 to transform the scale to a percentage-based scale, ranging from 0% to 100%. A transferred score of below 50% reflects poor well-being, and a 10-point change in the translated score is seen as clinically relevant [17]. Owing to its ease of use and high validity, the WHO-5 has been extensively used in mental health– and physical health–related mobile apps [19-23].

### Intervention

#### Overview

Users can choose to interact with Kai.ai through common messaging apps. Each user’s first interaction starts with an onboarding process where the WHO-5 is administered. Based on the users’ responses, Kai.ai builds 3 routines for the morning, noon, and evening. Each routine includes one or more of the service’s main blocks: (1) *Reflections and Journaling* and (2) *Exercises*.

#### Reflections and Journaling

For *Reflections and Journaling*, users are invited to think and write about their experiences. To maintain structure and help guide users, Kai.ai uses three kinds of journaling cues: gratitude, learning, and one good thing that happened.

During the gratitude exercise, users are encouraged to identify things for which they are grateful and to list them in writing, where they can easily review this list daily—this gives room to develop flexible thinking patterns. In the learning exercise, users are asked to focus on the lessons they can learn from their experiences and, lastly, in one good thing that *Exercises* users learn to reduce stress and anxiety by focusing on a single task rather than a long to-do list and spreading themselves too thin. Adopting a routine reflection and journaling process is meant to help users achieve a more centered, grounded, joyous, and purposeful state of mind.

#### Exercises

Two main types of exercises are offered to users: breathing exercises and ACT training. The breathing exercises focus on teaching users how to breathe in a constructive manner; breathing through their noses, using their diaphragm, and noting their posture. These are all meant to ensure a better flow of oxygen to the body, allowing for a reduction in stress and anxiety through the operation of the parasympathetic nervous system [24].

The used ACT training is based on the mindfulness approach aimed at increasing psychological flexibility, and treating pain and discomfort as facts of life can be used for personal growth through a process of acceptance and validation [16].

### User Interaction

Users have 2 main ways to interact with Kai.ai: passive and active. In the passive way, the service checks in on the user at least once and not more than 3 times a day, depending on the routine created during onboarding. The check-in prompts can include either *Reflection and Journaling* open-ended questions or closed questions where users are asked to indicate how happy they are. In addition to the passive check-ins, users can actively reach out to Kai.ai by simply texting it whenever they feel the need to.

### Data Analyses

To test Kai.ai’s impact on participants’ well-being, 2 analytical methods were used. First, a paired samples *t* test was performed to compare participants’ baseline WHO-5 scores, which were collected just before the initiation of use, and the last WHO-5 score recorded for each participant. Following the initial analysis, hierarchical linear modeling (HLM) was used to examine the change in the users’ well-being over time. HLM was chosen over other repeated measures models, such as repeated measures ANOVA, owing to its unique ability to model incomplete or imbalanced data sets. In the first level, the HLM was modeled to estimate the individual linear slopes and intercepts, and in the second level, the sample’s average slope and intercept were estimated.

## Results

### Paired Samples t Tests

**Figure 1:**
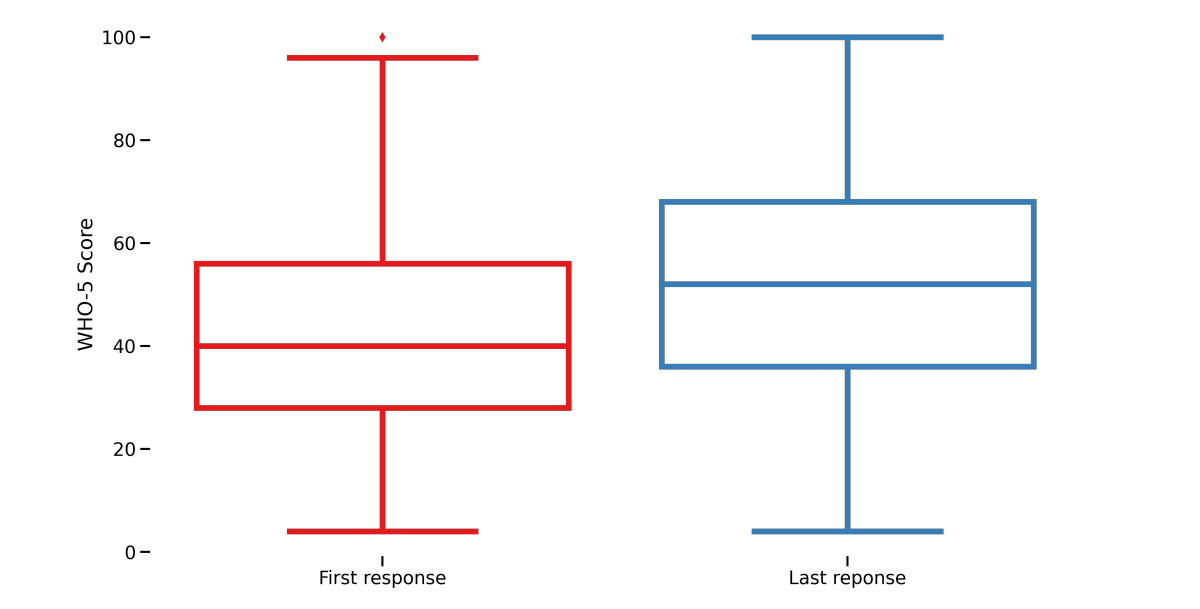
Box plot of the median (IQR) values of change in World Health Organization-Five Well-Being Index (WHO-5) scores for users' first and last measurements.

Users' scores on the WHO-5 were compared from the first and last measurement of each user. On average, users’ last measurement score was better (median 52) than their first (median 40). A Wilcoxon signed rank test indicated that this difference was significant (W=2682927; *P*<.001). Figure 1 shows a box plot of the median (IQR) values of change in WHO-5 scores.

### HLMs

In addition to the paired samples *t* test that was used to compare users first and last measurement scores, an HLM was used since it is best suited to deal with incomplete data sets while still accounting for the data set’s repeated measures nature [25]. This unique strength of the HLM analysis was this analysis because the number of measurements among users varied between 2 and 9. Some users used the app for only 2 months, others used it for well over a year.

Consequently, our model tested whether differences in users’ WHO-5 score between any 2 consecutive measurements could be predicted by the level of engagement during the time frame. As such, we used 2 variables as indications of engagement: the number of unique days during which the user had active interactions with Kai.ai during the specified time frame and the number of daily messages the user sent during that time. Using the R nlme package [26], the effect of the engagement variables on the difference in WHO-5 scores was first tested separately for each measurement time point (second to ninth) and then aggregated over all time points. Our results show that the difference in WHO-5 scores was linearly related to the number of daily messages a user sent (β=.029; t_81.36_=4; *P*<.001) and the interaction between the number of messages and unique number of days (β=–.0003; t_81.36_=–2.2; *P*=.03), but not to the unique number of days on its own (Table 1)

**Table 1:** Unconditional growth model for changes in the World Health Organization-Five Well-Being Index (WHO-5) scores over time.

WHO-5 score	Estimate (β)	SE	*t* test (*df*)	*P* value
Intercept	.563	1.045	0.536 (81.36)	.59
Total daily messages	.029	.007	4 (81.36)	<.001
Unique active days	–.015	.036	–0.427 (81.36)	.67
Interaction	–.0003	.0001	–2.2 (81.36)	.03

## Discussion

### Principal Findings

In this paper, we report the results of a pragmatic retrospective study aiming to test the effectiveness of a mobile phone–delivered, ACT-based, artificial intelligence–delivered conversational coaching platform. In the onboarding measurement, half of all participants reported a WHO-5 score of 40 or less, which is well below the cut-off point of 50, and an indicator of poor well-being. However, on the last measurement recorded for each user, half of all participants indicated a score of 52 and above, indicating a change for the better and an overall good well-being. These results highlight the great potential ACT-based mobile apps can have for improving users’ daily well-being.

### Limitations

This paper describes the use of a novel platform and treatment protocol using mobile messaging apps to deploy ACT-based treatment to improve the well-being of users. Our results, while highlighting Kai.ai’s great potential as reflected by the pre-post results, also hints at the wealth of possibilities still left to discover. The results of our HLM model, especially those of the negative interaction between the engagement variables, indicates that further research is needed to fully understand and describe Kai.ai’s potential and fit. An example of such an area, which future research should expand on, is Kai.ai’s suitability to different genders and age groups. A recent study found differences among these groups in the adaptability of mobile-based coaching tools, as females and younger age groups (18-25 years) showed higher adaptability than males and older ones, respectively [27]. To overcome these adaptability challenges, a number of key features are introduced, which are directly aimed at improving users’ engagement, leveraging the motivations of choice and progression. As such, an emphasis was placed on greater choice, as users are given the option of choosing tracks that focus on different central core ideas, such as relationships, mood, and life habits. Furthermore, over time, the daily routines and exercises offered to the user are selected on the basis of past preferences. Lastly, by allowing users to review their progress, allowing users the ability to view their progress time.

### Conclusions

The findings reported in this paper show Kai.ai’s great promise in helping individuals improve and maintain high levels of well-being and thus improve their daily life. These findings are further emphasized by the great need for mental health interventions, while the availability of and accessibility to treatment remains limited. It seems that the use of such technology, which is found to improve mental well-being per the findings of the WHO-5, is essential, especially nowadays.

## Abbreviations

ACT: Acceptance Commitment Therapy
HLM: hierarchical linear modeling
WHO-5: World Health Organization-Five Well-Being Index
